# The Progress of Smart Elderly Care Research: A Scientometric Analysis Based on CNKI and WOS

**DOI:** 10.3390/ijerph20021086

**Published:** 2023-01-07

**Authors:** Xiaoyun Liu, Ka-Yin Chau, Xiaoxiao Liu, Yan Wan

**Affiliations:** 1 Faculty of Business, Hunan First Normal University, Changsha 410205, China; 2 Faculty of Business, City University of Macau, Macao 999078, China

**Keywords:** smart elderly care, literature, scientometric analysis, CiteSpace

## Abstract

To reduce the burden caused by an increased elderly population and to provide efficient service resources, scholars worldwide have proposed and applied smart elderly care. This paper summarizes the hotspots of the existing literature and explores the research frontiers to ignite future research. CiteSpace software was used to conduct a scientometric analysis of high-quality literature collected from both the China National Knowledge Infrastructure (CNKI) and the Web of Science (WOS). Based on the results of the basic situation description, this article highlights six research hotspots in CNKI and 11 research themes in WOS. In addition, it offers three major evolution stages and three future research directions for smart elderly care research. This paper provides a holistic overview of the smart elderly care literature from two major global databases. The results will contribute to healthcare policy designers, practitioners, and developers by giving them comprehensive knowledge and generating strategies to enhance elderly people’s quality of life.

## 1. Introduction

Virtually every nation has experienced growth in its elderly population over recent decades [1]. According to a report from the United Nations, by 2050 there will be 1.5 billion people aged 65 years old in the world, which is likely to reach one sixth of the total population [2]. This continuous increase in the ageing population will bring an imbalance within the elderly care industry structure in many countries [3,4,5]. In the coming decades, several countries will face the dilemma of medical resource shortages and rising demand for nursing care for seniors [6]. As a possible way to deeply develop the elderly care industry, smart elderly care, an innovative ageing care mode based on disruptive internet technology, has received attention from scholars and experts [7,8,9].

Smart elderly care has grown rapidly as it is an effective way to solve the imbalance between the requirement of service and the supply available within the elderly care industry [8]. It is a service for seniors providing networking based on intelligent technology, which can satisfy the distinctive requirements of elders and bring dignity to seniors to achieve independent living. In the United States and European countries, smart elderly care has been applied in healthcare since 2007 to improve the well-being of elder people [8]. In China, it has increased breathtakingly since the state council published the guidelines of “Internet+” in 2015. According to the “Smart Ageing Industry White Paper” [10], the smart elderly care industry in China experienced steady growth from 2017 to 2019, whose market size increased from RMB 22 trillion to RMB 32 trillion.

Studies of smart elderly care in recent years have covered a wide range of research issues. Global scholars undertake their research from various perspectives such as the development history of smart elderly care, innovative service mode, vital technologies to assist senior care, etc. [11,12,13,14]. First of all, the development process of smart elderly care has been widely discussed. For example, Chopik and Bruggencate et al. [15,16] identified “social technology” as the theoretical motivation for applying smart elderly care. Then Meng et al. and Zuo [8,17] discussed the definition, opportunities, and challenges of the smart elderly care industry, reaching a consensus that the future of this industry is tremendously bright with the support of national policies. Thus, researchers have discovered several effective and reliable service modes from the perspective of smart elderly care [18,19]. For example, Sun and Zhang [19] proposed an “Internet+” model of smart elderly care service, which incorporates mobile phone Apps, service providers, products/services, the seniors and online platforms as an integrated system. Zhang [18] established an optimal model for smart ageing by combining the “visual business operation model”, the “intelligent public welfare matching model”, and the “integrated ecological co-construction model”. In addition, many researchers have used distinctive intelligent sensors and data-collection devices embedded in emerging technologies [3,7,20] to identify effective solutions to enhance the efficiency of ageing services and to minimize operational costs. Moreover, authors such as Neuhuettler et al. [9] have proposed a theoretical framework of service quality in the field of smart elderly care. Xu, Zhang, and Wei [11] displayed an indicator system to evaluate the service quality of smart community senior care to prompt service standardization and to reduce the differences in service levels among regions.

Although academics have contributed considerable intellectual work in this area, a comprehensive and holistic record of the smart elderly care literature containing both Western and Eastern views is lacking. Due to the distinctive historical and cultural background, political system, basic national conditions, etc., countries have different levels of ageing, which lead to particular problems and distinctive solutions. The Western world started the research on smart elderly care first, and their classic models are of reference value to developing countries. Thus, exploring the intellectual works in developed countries can help researchers to summarize the advanced experience and inspire others to discover their own path of improvement. Furthermore, as one of the biggest developing countries, China has the largest number of seniors in the world [8]. Many Chinese scholars have explored smart elderly care; however, their achievements have been overlooked because of language difficulties. Thus, it is problematic to accurately summarize global research on smart elderly care by analyzing only the English literature. As we know, the Web of Science (WOS) almost covers the most comprehensive and authoritative scientific literature worldwide [21], whereas China National Knowledge Infrastructure (CNKI) is the largest continuously updated database of Chinese academic literature; the CNKI effectively complements the English language databases and provides a comprehensive picture of the state of research in China. Hence, this study systematically compares the relevant literature on smart elderly care in both Chinese and English databases to present a holistic overview of current studies.

This paper explores the current global trend of smart elderly care by addressing two questions: What are the differences in the focus of research between English databases and Chinese databases? What is the future direction of smart elderly care? The outcome of this research could be useful to academics who intend to continue exploring smart elderly care in the future by presenting a holistic view of smart elderly care research from both English and Chinese databases. It may also inspire practitioners who want to minimize the cost and select a relatively effective service delivery method to enhance the life quality of ageing people via absorbing the advanced experiences from both developed and developing countries.

This article is organized as follows: Section 2 gives the data sources and research methods of this study, Section 3 analyzes the English literature, while Section 4 describes the Chinese literature. The comparations and hotspots investigation is presented in Section 5. Section 6 illustrates the future directions. The last section is the conclusion.

## 2. Literature Review

Smart elderly care (smart/intelligent senior care) was first introduced by Unit Trust UK, and it refers to providing ageing services via adopting information technology and intelligent control technology [4]. Zuo [17] emphasized that its core concept is building a technological network for senior care service participants including government, communities, healthcare institutions, doctors and nurses. Thus, the research of smart elderly care incorporates various perspectives from distinctive disciplines such as information engineering, public management, health and medical care, etc.

As smart elderly care can be explored in different academic areas, the literature review displays an extensive and disorganized picture. Firstly, a considerable amount of published literature reviewed the technologies adopted in smart elderly care. For instance, Marques et al. [22] conducted a review on eHealth technologies for older person’s outdoor spaces rehabilitation. Kong et al. [23] summarized the application of mHealth technologies for elderly people care in developed countries. Moreover, the utilization of some specific information technologies, such as Artificial Intelligence and the Internet of Things, are extensively discussed in the field of smart elderly care. For example, Shaikh et al. [24] presented Extended Reality (XR) technology for elder people to acquire remote consultation with doctors. Tun et al. [3] provided a systematic review of IoT and wearable technologies adopted in elderly healthcare and presented new research opportunities in the emerging field such as robot technology. Secondly, the review of smart homes for older adults is another main topic. For instance, Sepasgozar et al. [25] reviewed the application of IoT technologies in the smart home. Zhang et al. [18] displayed the development process of the smart home for elderly care in China and identified the deficiencies and further efforts to optimize the improvement. Finally, some literature reviews from the perspectives of sociology and ethics are also relevant. For example, Grossi et al. [26] analyzed the framework of positive technology adopted in elderly care, and discussed the gap between theoretical requirements and system availability. Zhang et al. [27] identified the major ethical issues when applying smart elderly care, and emphasized the necessity of considering these ethical conflicts.

The existing bibliometric literature therefore mainly analyses certain aspects of smart ageing and does not provide a comprehensive analysis of the overall state of the field. In addition, the source of this literature is an English language database. Therefore, in order to obtain more comprehensive results, we have added Chinese literature to the existing English literature, using the WOS and CNKI databases as sources to systematically discuss research trends in the most important databases on smart ageing.

## 3. Research Methods

### 3.1. Data Sources

The Web of Science (WOS) is the world’s most trusted publisher-independent worldwide citation database, while the China National Knowledge Infrastructure (CNKI) is the largest and most influential database in China [21]. To ensure representativeness and accessibility, we selected WOS as the effective source of data retrieval for English literature, and CNKI as the data origin for Chinese papers [28]. According to Professor Zuo, the founder of Smart Ageing Institute in China, “ZHI HUI YANG LAO” is an inherent noun which should be translated into “smart elderly care, smart care for the aged or smart senior care”. Before the year 2016, “ZHI NENG YANG LAO” which is translated into “intelligent elderly care”, is widely used in China [29]. Thus, in order to acquire an accurate comparison of the literature with the same research themes from English and Chinese databases, we used TS (Topic Search) “smart elderly care” or “smart senior care” or “intelligent elderly care” or “intelligent senior care” or “smart ag*ing care” or “intelligent ag*ing care” as the search terms, and the year span was set to all years. The data were collected on 19 July 2022. Some 2591 studies were obtained from WOS. For CNKI, with no time limit, searching with “smart elderly care (ZHI HUI YANG LAO)” or “intelligent elderly care (ZHI NENG YANG LAO)” as the subject produced a total of 3621 results.

To extract the most relevant and highly valued academic articles, we set up advanced restriction criteria. For CNKI results, we excluded newspapers, books, projects, and older papers. The specific selection criteria were Journal = “North Core” + “CSCD” + “Chinese Social Science Citation Index (CSSCI)”. Thesis = doctoral academic master thesis. After screening via article content, we retained 327 academic works for further study. For WOS, the restriction strategies were Document type = article; Database = SSCI and SCIE. According to Yao et al. [30], only peer-reviewed articles can present original scientific improvement, and the selection of a database is necessary to ensure the high quality of research articles. This was followed by a manual approach to data cleaning, mainly by merging near-sense words and removing generic words to avoid affecting the analysis results. Some 560 articles were maintained for the next analysis. It was necessary to emphasize that after the duplicate selection procedure from CiteSpace, only 504 papers remained.

### 3.2. Research Methods

Scientometric analysis is a relatively effective method for objectively illustrating a scientific knowledge map and identifying the major research themes [31]. It can not only reveal the sources of knowledge and its development pattern, but also graphically express the structural relationship and evolution pattern of knowledge in related fields [32]. We conducted scientometric analysis in this research, which included yearly distribution, major research institutions, co-authors, and keyword co-occurrence. These approaches are statistical and cluster analyses of literature keywords, subject terms, etc., using mathematical algorithms and econometric methods to obtain hot topics and cutting-edge trends in one field [33].

We used CiteSpace for the scientometric analysis. According to Chen and Zhang et al. [21,34], CiteSpace is a java-based visualization program developed by Dr. Chen Chaomei which can scientifically analyze the knowledge in literature extracted from WOS and CNKI, and which displays graphs based on quantitative analysis [35]. It is capable of drawing co-occurrence maps of authors, institutions, keywords, etc. In addition, the software has unique advantages in terms of clustering techniques and mapping [36]. CiteSpace focuses on connecting lines to present the strength of each topic, and it deconstructs the clustering relationships between nodes. Thus, this software can accurately explore the essence of the research topic.

**Figure 1:**
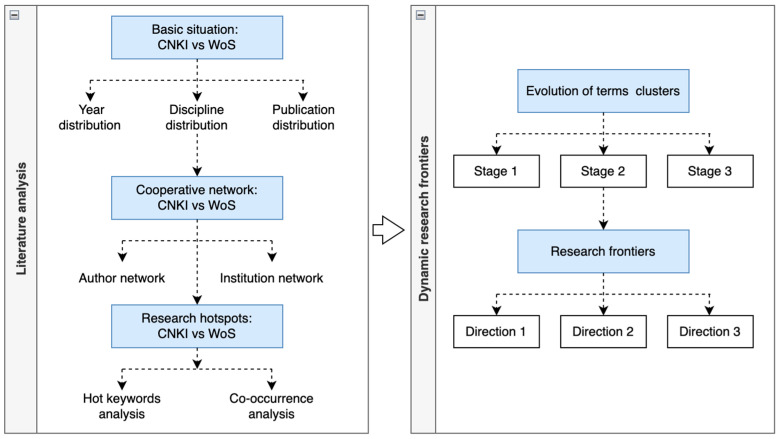
The process of this research.

The major analysis procedures were as follows: First, we inputted the data from each database in plain text format into CiteSpace to generate the project and to set up the parameters for each visualization tool. Second, we recognized the basic situation of current research by exploring the publication distribution and discovering the cooperation among authors and institutions. Third, we constructed the knowledge base by identifying the most cited journals related to smart elderly care. Fourth, we used co-occurrence analysis to identify the major research themes, and we performed burst term analysis to discover the frontiers of smart elderly care. Fifth, we distinguished the differences between WOS and CNKI and illustrated future hot topics by integrating the results of two databases. The specific process is displayed in Figure 1 [33].

## 4. Analysis of the Literature

This section presents the analysis results from a basic situation, cooperation network, and research hotspots via comparing literature from CNKI and WOS.

### 4.1. Basic Situation

#### 4.1.1. Year Distribution

**Figure 2:**
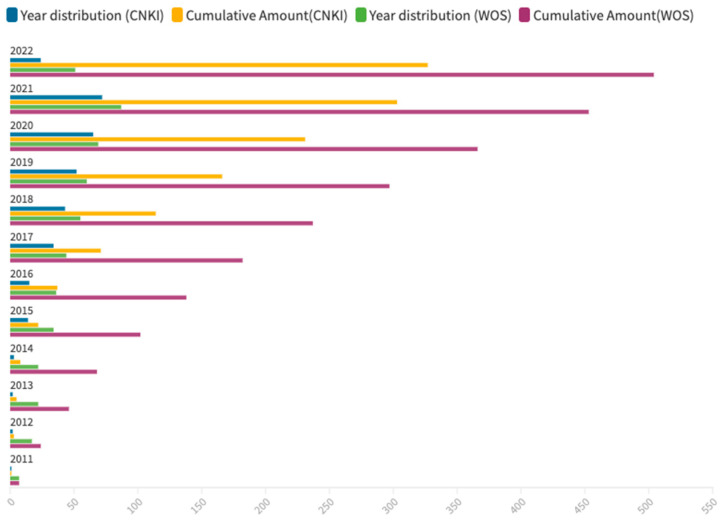
Publication distribution of CNKI (retrieved on 17 July 2022) and WOS (retrieved on 19 July 2022).

According to the statistical results of CNKI, the amount of literature focused on smart elderly care is increasing dramatically. Figure 2 illustrates the total of 327 highly valued Chinese publications between 2011 to 2022. It shows that the history of Chinese scholars studying smart elderly care is relatively short. There were only 37 articles published from 2011 to 2014, which accounts for just 11.31% of the total, while the rising stage of smart elderly care started in 2017 when *the Action Plan for the Development of Smart Health and Elderly Care Service Industry (2017–2020)* was published by the State Council of China. The number of intellectual works then rose to 7.84 times what it was at the end of 2016.

Figure 2 also shows that the research on smart elderly care from WOS also started in 2011. The publication distribution of academic papers from WOS witnessed a relatively steady rising trend from 2011 to 2022. The period from 2011 to 2014 was the initial stage, in which the proportion of publications was 13.50%. During this stage, the concept of smart elderly care was introduced and accepted, and this built a theoretical base for practical development. The next five years (2015–2020) were a continuously increasing stage, and they accounted for 59.12% of the publications. This phase focused on application, particularly innovative designs and research based on information technologies and multi-disciplinary investigations. Therefore, this research gradually matured during this phase. By 2021, the growth rate of published studies had risen to 26.09%, which was twice that of 2020, making it likely that there would be significant growth in research in smart elderly care from 2021.

#### 4.1.2. Distribution of Disciplines

Table 1 displays the distribution of disciplines of the academic articles from CNKI, which reveals that social science researchers paid more attention to this field. It shows that national politics is the major subject of Chinese literature, as it occupied 41.28% of the related studies. It was followed by the topics of trading economics and service economics, which reached 10.70% and 10.40%, respectively. Other disciplines such as medical policy, information economics, social science and statistics, computer science, clinical medical, automation technology, administration, etc., made up the remainder.

The disciplinary distribution of papers from WOS (Table 2) presents a completely different scenario than that of CNKI, as the top 10 subjects all belong to natural science. Among the total of 504 articles, 191 come from computer science and another 191 come from engineering, which both accounted for 34.11%; telecommunications, health care science services, and instrumentation are also vital disciplines in this research.

#### 4.1.3. Publication Disciplines

Table 3 summarizes the top 10 publications from CNKI. The results show that four articles discussed the service model of smart elderly care, and another four papers revealed the obstacles, characteristics, policies, and development of smart elderly care. The last two publications concerned the information technologies adopted in smart elderly care; the authors presented the dilemmas and explored the solutions, respectively. All 10 articles used theoretical research, which indicates that Chinese research on smart elderly care focuses on qualitative methods and a macro view.

Table 4 displays the 10 most frequently cited articles from WOS. By contrast, they all concentrated on the technology development of smart elderly care. Portet et al. [37] discussed technology acceptance in smart elderly care. Rashidi and Mihailidis [38] summarized the technologies, tools, and techniques of ambient-assisted living. Robinson et al. [39] reviewed the factors that impact admission to nursing care, and they identified the role of health robots as a solution to meet seniors’ requirements in home care. The other seven papers focused on the design and application of smart elderly care technology improvement.

### 4.2. Cooperation Networks

#### 4.2.1. Author Networks

**Figure 3:**
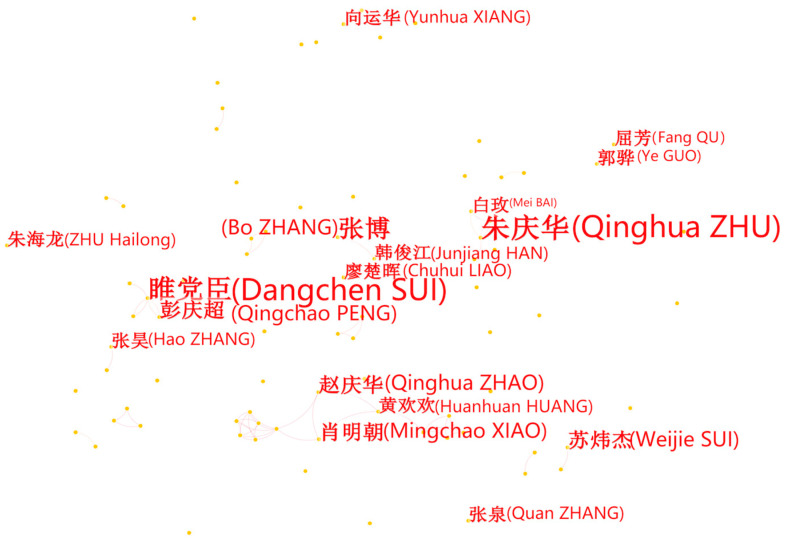
The author networks of studies from CNKI. (The contents in brackets are the translations of the original results which are presented in Chinese).

The analysis of the author cooperation network revealed that 585 authors have explored smart elderly care, of whom 17 have published more than three authoritative journal articles. Xinxu Wu and Zhuojing Ding, who discussed the prospect of new media to optimize the lifestyle of the elderly in 2011, were the earliest authors in this field [40]. The left side of Table 5 displays the top 10 authors based on the publication number via CiteSpace. Figure 3 illustrates the co-author network of smart elderly care studies selected from CNKI. There were four major co-author clusters: the first was Group “Dangchen SUI (count = 8) and Qingchao PENG (count = 4)”. The second was Group “Qinghua ZHU (count = 8) and Mei BAI (count = 3)”. The third was Group “Bo ZHANG (count = 6) and Jiangjun HAN (count = 3)”, followed by the fourth group “Qinghua ZHAO (count = 4), Mingchao XIAO (count = 4), and Huanhuan Huang (count = 3)”. Moreover, eight of 10 authors listed on the left side of Table 5 were group members or group leaders of the co-author clusters above. For the Chinese database, there was no international cooperation in this field.

**Figure 4:**
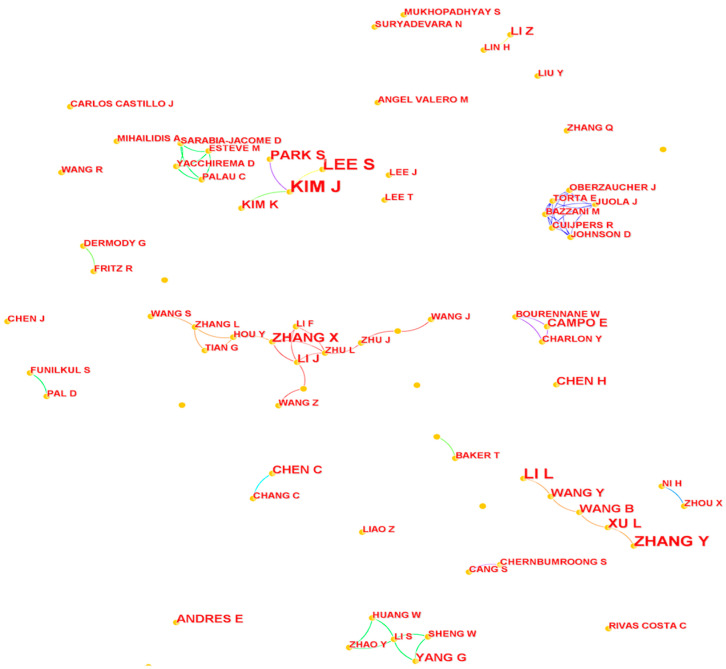
The author network of studies from WOS.

The right side of Table 5 illustrates that the publication number of top authors in WOS is similar to that of CNKI, and that Asian scholars pay more attention to relevant research. In addition, Figure 4 displays three major author networks. The first cooperative group refers to KIM J, LEE S, PARK S, and KIM K, three of whom are listed in the top 10 authors on the right of Table 5. The next group is displayed in the lower-right corner of Figure 4, and it contains members such as ZHANG Y, XU L, LI J, WANG B, and WANG Y. Three of them also appear in the top 10 authors table. Last, the biggest cooperative group is in the middle of Figure 4. ZHANG X, who was ranked as the fifth most important author, has an intimate partnership with 10 other scholars.

#### 4.2.2. Institution Networks

**Figure 5:**
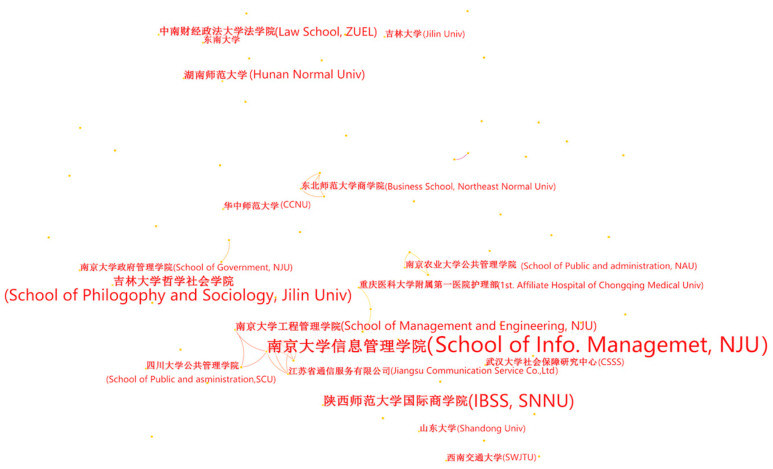
The institutions’ networks of studies from CNKI. (The contents in brackets are the translations of the original results which are presented in Chinese).

As Table 6 and Figure 5 show, Nanjing University (NJU) was the major research institution which not only conducted collaborations between internal schools—such as the partnership between the School of Information management and School of Management and Engineering—but also organized cooperation with other universities such as Sichuan University (SCU) and Jilin University. In addition, NJU is also associated with the Jiangsu Communication Service Company for university–enterprise cooperation in scientific research. However, the internal university scientific groups were more prevalent. For example, Shaanxi Normal University (SNNU), Hunan Normal University, Shandong University, and Southwest Jiaotong University (SWJTU) preferred domestic collaboration. In addition to universities and research institutions, hospitals such as the First Affiliate Hospital of Chongqing Medical University showed an interest in smart elderly care as well.

**Figure 6:**
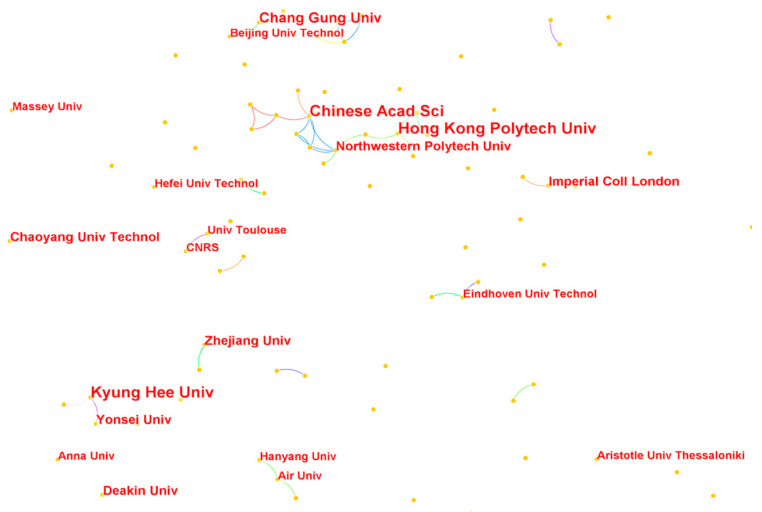
The institution network of studies from WOS.

Table 7 displays the research institutions with high productivity of academic papers. Eight out of 10 are Asian universities, which is consistent with the analysis of the author network that Asian academies are more concerned than others about smart elderly care. However, in recent years, Western institutions such as Deakin University and Imperial College, London have also shown their interest in this research. Furthermore, Figure 6 illustrates the cooperation cluster among institutions, and it also reveals three obvious clusters. The first one is Hong Kong Polytechnic University, the Chinese Academy of Science, and Northwestern Polytechnic University. The second one refers to the cooperation between Beijing University of Technology and Chang Gung University from Taiwan. The last one is the cluster from Korea, which indicates the collaboration between Kyung Hee University and Yonsei University.

### 4.3. Research Themes Analysis

#### 4.3.1. Hot Keywords Analysis

Hot keywords are subject terms that are of high interest to scholars and that recur in the literature within a specific time [41]. By analyzing hot keywords, we can grasp the hot topics and developments in the research field. In this paper, the frequency and centrality of keywords in the literature on smart elderly care citation are calculated with the help of CiteSpace software.

Table 8 extracts the major terms used in smart elderly care both from CNKI and WOS. The high-frequency keywords from CNKI reveal the research hotspots, which can be summarized as smart elderly care, smart pension, population ageing, smart home and information technology. Smart elderly care includes the keywords smart elderly care (count = 16, centrality = 0.19), smart senior care (count = 6, centrality = 0.05), and elderly care service (count = 9, centrality = 0.06). The smart pension cluster contains the terms smart pension (count = 16, centrality = 0.23), intelligent pension (count = 6, centrality = 0.05), and pension industry (count = 4, centrality = 0.01). Smart home consists of smart community (count = 4, centrality = 0.02) and home-based care (count = 4, centrality = 0.02). Information technology incorporates artificial intelligence (AI) (count = 12, centrality = 0.23), big data (count = 7, centrality = 0.09), and information technology (count = 4, centrality = 0.01). The keywords went into the population ageing cluster.

The keywords from WOS show that the research hotspots are smart elderly care, smart home, information technology, and ambient intelligence. The smart elderly care cluster focused on the adoption (count = 22, centrality = 0.09) of elderly care (count = 70, centrality = 0.17). The smart home cluster contains technology-development issues such as activity recognition (count = 50, centrality = 0.07) and fall detection (count = 18, centrality = 0.10). The terms from WOS also showed that topics relating to information technology are the most trending ones, for instance, the IoT (count = 52, centrality = 0.08) and deep learning (count = 15, centrality = 0.03). The ambient intelligence cluster contained keywords such as ambient assisted living (count = 53, centrality = 0.16), etc. To acquire a more specific view of hot research themes, it is necessary to construct a visualization cluster of co-occurrence terms.

#### 4.3.2. Co-Occurrence Terms Analysis

Although the analysis of high-frequency keywords can give a general idea of the dynamic development of smart elderly care, the links between the hot terms and the specific aspects of smart elderly care research mapped through the links were further investigated with the help of CiteSpace software.

##### Research Theme Analysis of Studies from CNKI

**Figure 7:**
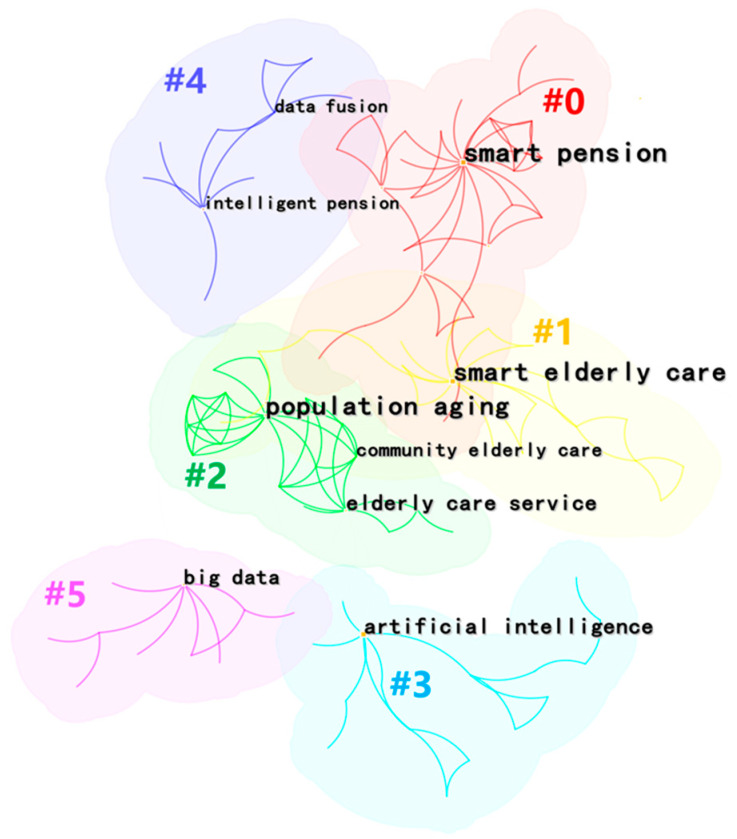
Cluster of co-occurrence terms.

To understand the research hotspots of smart elderly care further, we conducted a co-occurrence analysis and we acquired six clusters (Figure 7). The Density = 0.0106, Modularity Q = 0.8633 (larger than 0.3), and Mean Silhouette = 0.9619 (larger than 0.5), which represents the validity and reliability of the clustering procedure [42]. According to Figure 7, the major hotspots of smart elderly care can be categorized into seven clusters.

The smart pension (#0) cluster covers issues of smart elderly care and pension finance. Chen and Mei [43] established an intelligent pension system based on IoT technology, analyzed the application of IoT in Fujisawa Sustainable Smart Town (Fujisawa SST), and presented suggestions for elderly care architecture in China. Hao, et al. [44] explored the new public–private partnership model for the elderly to innovate the financing model of the old-age service industry and to increase the effective supply of services.

The smart elderly care service (#1) is one of the main research themes that includes the exploration of elderly care mode and the demand for senior care. According to [17], there are four different modes of smart elderly care in China: smart community, smart home, smart institution, and smart virtual. Sui and Peng [45] analyzed the obstacles of traditional home care and recommended solving the problems with information technology.

The population ageing (#2) cluster mainly discussed the situation and policies relating to ageing people, which proposed introducing a disruptive ageing care mode and raising people’s attention to smart elderly care. For instance, Zhai et al. [46] described the trends, characteristics, and relevant policies of elderly care, and they elaborated on the possibility of applying smart elderly care in China.

The fourth research theme is AI (#3), which involves wisdom endowment, family pensions, and elderly service law. Zhang and Song [47] showed that information technology will provide a revolution in the field of elderly care. Thus, building the standard of smart elderly care service, promoting smart elderly care, etc., are effective ways to improve the scientific innovation of the elderly care industry.

The next cluster is data fusion (#4), which covers topics around the integration of medical care and pensions. Scholars are committed to integrating nursing care into elderly care together with innovative information technology [48].

The No. 6 research hotspot is big data (#5), which can suggest senior care models and covers the practical significance of the smart city. IoT and wearable sensor equipment are mentioned in this topic. Pan and Song [49] stated that community elderly care is an effective way to solve the difficulties in ageing care in China. They also emphasized that constructing a smart home can prompt the sustainable development of community ageing care.

##### Research Theme Analysis of Studies from WOS

**Figure 8:**
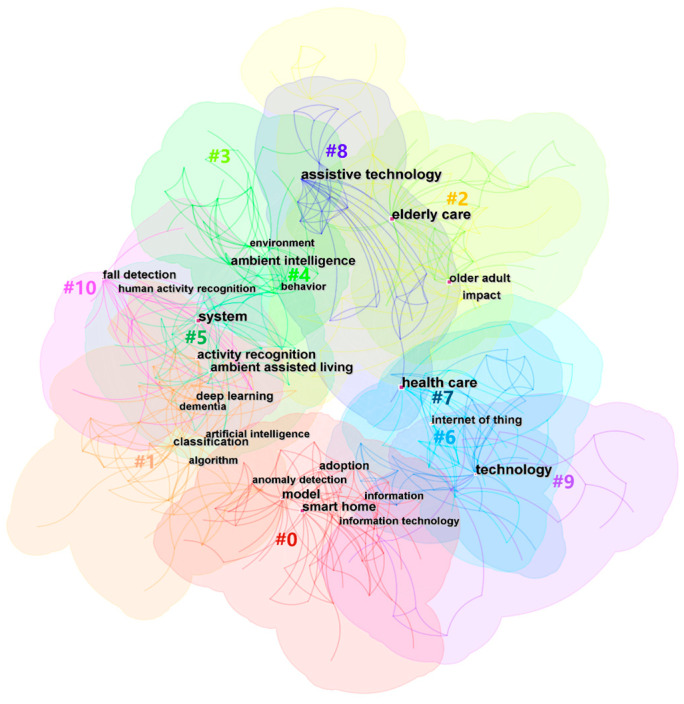
Hot spot cluster of studies from WOS.

As the cluster of co-occurrences of keywords illustrated, the major hotspots of smart elderly care from WOS are gathered closely together. This effect can be described by the following 11 clusters based on Figure 8. The Density = 0.0129, Modularity Q = 0.5862 (larger than 0.3), and Mean Silhouette = 0.8295 (larger than 0.5), which implies the validity and reliability is suitable [42].

Smart home (#0) is the main research theme, and it included the technology acceptance of smart home modes and the investigation of anomaly detection. For instance, Portet, Vacher, Golanski, Roux and Meillon [37] conducted interviews to determine older people’s acceptance of the voice interface in a smart home. It addressed the conclusion that the voice interface seems to have great potential to ease the daily life of senior people. Zhang et al. [18] reviewed the different development stages of smart homes and suggested the future directions of smart home research, such as integrating public and private smart home platforms and developing standards for smart elderly care technology.

This is followed by the machine learning cluster (#1), which contains technical keywords such as AI, algorithms, and classification. Other topics in this cluster are connected with AI technology, including deep learning and artificial neural networks. For example, Hossain and Muhammad [50] adopted healthcare IoT technology to monitor and analyze complicated health data from the increasing number of elder people and patients.

The service quality (#2) research theme covers topics around elderly care service and the impact of technologies on elderly service. It also concerns topics such as the elderly living alone and topological mapping. Xu, Zhang, and Wei [11] constructed an evaluation system for the service quality of smart elderly care based on a two-stage decision model, which can reduce the difference in service levels among varying districts.

The next two clusters are ambient assisted living (#3) and ambient intelligence (#4), for which most of the areas are overlaid in Figure 8. However, the former emphasized the behavior of smart elderly care and the latter focused on the intelligent environment. Assisted living (#5) covers topics related to activity recognition, indoor localization, and other assisted modes to develop relevant intelligent systems for smart life. Big data (#6) explored assisted living technology such as the IoT, cloud computing, fog computing, etc. For instance, Rashidi and Mihailidis [38] described the use of ambient assisted living tools under the pressure of the ageing population. They also summarized the current advanced technology and the future challenges. Campos et al. [51] reviewed articles relating to ambient intelligence and social networking sites which have an impact on senior care. They also identified the main technological resources and strategies to encourage ambient intelligence and social networking in ageing life.

The other clusters discussed the research theme of ageing care. The care delivery (#7) cluster focused on issues relating to health care, vital signs, health monitoring, and holistic health. Moreover, the ageing cluster (#8) investigated assistive technology for elderly care. Primary care (#9) covered research relating to older patients’ health care. The last one was fall detection (#10), which focused on investigating wearable technology related to physical activity. For example, Yu et al. [52] proposed a fall detection system based on computer visualization, which was used to monitor ageing people in home care. Suryadevara and Mukhopadhyay [53] reported on a wireless sensor linked with house appliances to assess the health function of ageing people and monitor the daily activities of seniors.

## 5. Dynamic Research Frontiers of Smart Elderly Care

Due to the dynamic of research hotspots, the development and evolution of research themes in smart elderly care tend to change over time. Therefore, this study compares the inheritance relationship of hot keywords in each period to analyze the evolution of research hotspots and to predict the research frontiers of smart elderly care via a timeline picture and a burst term analysis generated by CiteSpace.

### 5.1. Evolution of Term Clusters

**Figure 9:**
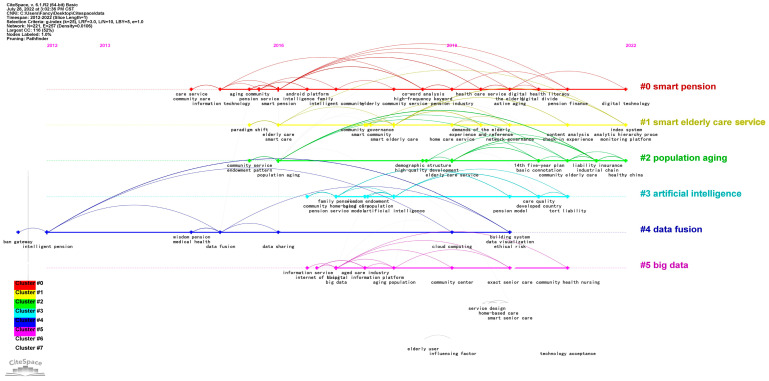
The research timeline of smart elderly care from CNKI.

**Figure 10:**
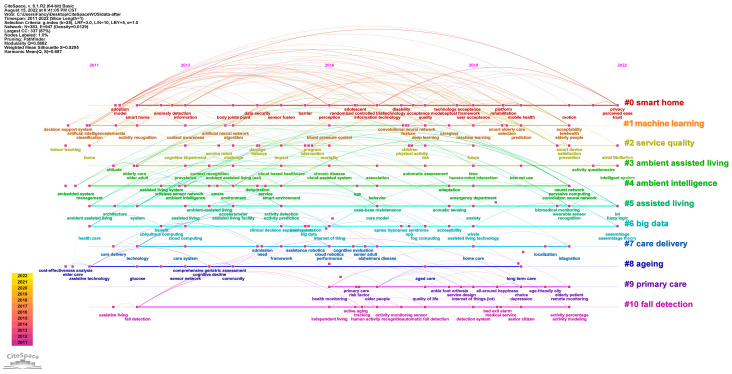
The research timeline of smart elderly care from WOS. Note: These figures were generated using the CiteSpace Software. Due to the size of the figure and page layout, the image may not be clear. For access to the high-quality version of these figures, please visit the following website: https://figshare.com/articles/figure/The_research_timeline_of_smart_elderly_care_WOS_and_CNKI_/21800783 (accessed on 25 December 2022).

Figure 9 and Figure 10 show the timelines of keyword co-occurrence of literature from CNKI and WOS, respectively. They reveal the major topics of smart elderly care and the duration of the topic’s importance [54]. By clustering the terms, we identified three different evolution stages of smart elderly care.

#### 5.1.1. Stage 1 (2011–2015): The Initial Research on Smart Elderly Care

According to Figure 9, only two clusters—smart pension (2014–2022) and data fusion (2012–2020)—cover the period of Stage 1. The smart pension cluster explored the various models of elderly care. It introduced smart pensions and the ageing community based on the information technology platform. The data fusion cluster began its research on selecting an elderly care model via integrating medical care into smart elderly care.

While 10 of the 11 clusters in Figure 10 were active at this time, the achievements in WOS are more plentiful than those in CNKI. Similarly, the smart home (2012–2022) cluster focused on the adoption model and development barriers during this period; the service quality (2011–2022) group emphasized exploring the impact and challenge of technological products in elderly care. The ambient assisted living (2012–2022), ambient intelligence (2011–2021), and assisted living (2011–2022) clusters focused on seniors’ attitudes towards ambient intelligence and assisted living technologies. The other clusters such as big data (2011–2022), care delivery (2012–2021), ageing (2011–2020), and fall detection (2012–2021) discovered the benefits and handicaps of care systems and assistive technologies.

In brief, scholars in Stage 1 identified the conflicts between demand and supply for senior care. To improve the efficiency of allocation, smart elderly care was introduced as a significant strength to ease these conflicts. Specifically, this may reflect the requirement for seniors to have wireless sensors; in addition, it can connect service providers, service receivers, and service monitors via a cloud platform [55]. Subsequently, scholars have been committed to exploring different models of smart elderly care. It has been shown that smart elderly care can be integrated with medical care, smart community, and the real estate industry [56,57].

#### 5.1.2. Stage 2 (2016–2020): The Development of Information Technology

According to Figure 9, this stage is the most flourishing phase, as it emphasized the development of information technology. The smart pension cluster moved its attention to healthcare service research in the literature via co-word analysis and high-frequency keywords analysis. Around 2019–2020, the data fusion cluster focused on cloud computing, system visualization, and ethical risks. The population ageing (2016–2022) cluster identified the ageing population as a national issue [58], and then it moved on to investigating the demographic structure and developing high-quality elderly care by enhancing technology. The smart elderly care service (2016–2022) cluster began with the arguments about a paradigm shift from traditional elderly care to smart care. With the introduction of smart community and community governance, the new format of community elderly care was discovered in 2017–2018. The AI cluster explored the techniques of AI and wisdom endowment used in community home-based care, family pensions, and pension service from 2016 to 2018. In 2021, it was concerned about how to change care quality and pension models with AI. There was also a discussion of tort liability when using AI technology. Over the same period, the big data cluster first investigated the information service involved with the IoT, big data, and information platforms. Then, it discussed how to use big data techniques to form community centers and eventually to conduct exact senior care for community health nursing.

Although all 11 clusters in Figure 10 appeared in this stage, the outputs are lower than in the last stage. The smart home cluster still focused on the technology acceptance model. Machine learning (2011–2021) explored deep learning technology for blood pressure control and neural networks. In addition, Clusters #3, #4, #5, and #6 were committed to studying varying forms of information technology such as the IoT, big data, cloud-assisted systems, acoustic sensing, etc. The last four clusters (#7, #8, #9, and #10) integrated the concept of smart city and home care to enrich smart elderly care. Thus, tracking, monitoring sensors, detection systems, and other remote technologies relating to long-term care were emphasized during this period.

In summary, the construction of a smart elderly care system relies on the arrangement of smart cities and smart communities. At the same time, modern information technology is the core support for smart ageing. This shows that throughout the research process of smart ageing, studies of technologies such as the IoT, cloud computing, big data and AI have never stopped, and they are becoming more mature as time passes.

#### 5.1.3. Stage 3 (2021-Date): High Quality Service for Smart Elderly Care

To achieve active ageing based on digital technology, the digital divide and pension finance became the most prevalent topics in the smart pension cluster. In addition, the smart elderly care cluster considered the demands of seniors and finally aimed to set up the index system to enhance the quality of service. The analytic hierarchy process is the most prevalent research method to construct the ratio system. The population ageing cluster adopted the idea of an industrial chain in the hope of achieving sustainable health in China. Figure 10 shows that the smart home cluster is concerned about privacy, perceived ease, and trust in smart elderly care. The ambient assisted living and assisted living clusters also explored the service quality of intelligent elderly systems based on fuzzy logic.

In a word, Stage 3 focused on enhancing the life quality of elder people from all perspectives. As China published its 14th five-year plan in 2021, a high-quality senior care industry was developed. Therefore, this stage involved discussion of the basic connotation of the policy and exploration of the industrial chain and liability insurance issues of community elderly care to present the picture of a healthy China. In addition, worldwide scholars focused on constructing the whole smart elderly care system, together with the inner and outside factors connected with smart elderly care. Thus, researchers mentioned ethical issues and the evaluation system for service quality of intelligent senior care to motivate the sustainable development of smart elderly care.

### 5.2. Analysis of the Research Frontiers

Although the cluster analysis of these dynamic terms can effectively clarify the evolution of smart elderly care over time, it seldom reveals valuable research directions or potential research fields [59]. However, Kleinberg’s burst detection algorithm can accurately detect the rapid growth of a specialized vocabulary in a short period. The burst terms identified by CiteSpace are the most trending keywords adopted in the studied literature, and they can be used to grasp and predict the research future via analyzing their characteristics and distribution [60]. In this paper, high burst keywords were acquired based on the burst detection technology provided by CiteSpace.

The burst terms from CNKI and WOS are in Table 9, and by combining the dynamic evolution clusters and burst terms, research frontiers for smart elderly care research can be predicted.

(1) Next-generation information technology

Scholars will focus their research on the technological breakthrough aspects of smart health, further strengthening the capacity to build up laboratories, engineering centers, and other research infrastructures. In recent years, the next generation of information technology, which refers to 5G, the IoT, cloud computing, big data, AI, virtual reality, etc., has been applied widely. Their position as basic technical supports in smart health is increasingly highlighted. Therefore, in future research trends, aspects such as technological breakthroughs and expansions of application scenarios in the development of smart health will also become the core of academic research [61].

(2) Home-based smart community elderly care

As the conflicts between supply and demand for elderly care have occupied a prominent position, home-based smart community elderly care is an effective way to integrate the resources to meet the requirement of the rising senior population. In addition, many policies encourage the combination of home care and community care to ensure the diversity of senior care resources [62]. Consequently, relevant research such as constructing smart platforms for home-based smart community care will become the research frontier, and it is also a research direction with great potential.

(3) Sustainable development of smart elderly care

How to develop a sustainable and harmonious smart elderly care industry will be a prevalent issue in the future. Intelligent technology, which has been widely discussed, can enhance the performance of smart elderly care. While contemporary scholars will conduct their research from many directions such as government responsibility, policies and regulations, financial and capital issues, etc., according to [63], ethical problems such as improving the privacy protection system and quality monitoring issues such as strengthening the standardization system will substantially support intelligent elderly care and significantly enhance its growth.

## 6. Conclusions

We collected 307 high-quality Chinese academic studies assembled by CNKI and 504 English research articles collected by WOS from 2011–2022 as research objects. We conducted scientometric analysis to identify the basic situation, cooperation networks, hotspots, and research frontiers of smart elderly care, and our conclusions are as follows:

First of all, the time distribution of publications from both CNKI and WOS witnessed a year-on-year increase. Compared with WOS, smart elderly care research in CNKI received less attention, and there were limited publications before 2016. However, after the release of the Action Plan for the Development of Smart Health and Elderly Care Service Industry (2017–2020) in 2016, there was a dramatic rise in publications. In brief, more scholars began to research smart elderly care, which implied the rapid development of this industry.

Second, the disciplinary distribution in CNKI focused on topics related to social science, such as national politics, trading economics, service economics, and administrative management; natural science only took up limited proportions. Nevertheless, the situation in WOS displayed a completely different view, in which the natural science disciplines occupied the major advantages. This phenomenon shows that scholars have explored smart elderly care from all angles and perspectives.

Third, from the studies of authors and institutions, both WOS and CNKI contain papers from research teams that were investigating smart elderly care. For instance, the SUI D and PENG Q teams were committed to exploring the smart elderly care model which can be adapted to Chinese society. In WOS, the ZHANG X and ZHANG Y teams focused on the application of information technology in smart elderly care. Compared to WOS, most of the research institutions in CNKI were business schools, and they were easily affected by district restrictions.

Fourth, only six hotspots were recognized in CNKI, while 11 clusters were identified in WOS. The research themes in CNKI focused on the general description of the current situation of smart elderly care in China, the exploration of the most adapted model, and superficial analysis of information technology application in smart elderly care. In contrast, the research topics in WOS illustrated a relatively holistic and profound perspective. In addition to the research themes mentioned above, they also covered service quality research, ethical issues, and specific analysis of related information technologies to ensure the sustainable and harmonious growth of smart elderly care.

Fifth, there are three major research frontiers: next-generation information technology, home-based smart community elderly care, and the sustainable development of smart elderly care. The improvement of smart elderly care needs the support of next-generation information technology, and it also requires the conversion and upgrading of service models and systems. Finally, no matter how smart elderly care becomes, it must take sustainable growth as its primary premise.

## Figures and Tables

**Table 1:** The distribution of the top 10 subjects of the studies from CNKI.

Rank	Subject	Quantity	Proportion
1	National politics	135	41.28%
2	Trading economics	35	10.70%
3	Service economics	34	10.40%
4	Medical policy	18	5.58%
5	Information economics	18	5.58%
6	Social science and statistics	13	3.97%
7	Computer science	12	3.67%
8	Clinical medical	10	3.06%
9	Automation technology	9	2.75%
10	Administration	4	1.20%

**Table 2:** The distribution of the top 10 subjects of the studies from WOS.

Rank	Subject	Quantity	Proportion
**1**	Computer science	191	34.11%
**2**	Engineering	191	34.11%
**3**	Telecommunications	91	16.25%
**4**	Health care sciences services	68	12.14%
**5**	Instruments and instrumentation	56	10.00%
**6**	Chemistry	54	9.64%
**7**	Medical informatics	52	9.29%
**8**	Physics	33	5.89%
**9**	Geriatrics and gerontology	28	5.00%
**10**	Public environmental occupational health	28	5.00%

**Table 3:** The top 10 publications in CNKI.

Rank	Title	Journal	Year	Citation Count
**1**	“Internet+ home-based care for senior citizens”: Research in the service model of intellectual home-based care for senior citizens	Journal of Xinjiang Normal University (Philosophy and Social Sciences)	2016	398
**2**	Aging in China: General trends, new characteristics and corresponding policies	Journal of Shandong University (Philosophy and Social)	2016	282
**3**	“Internet+ community-based senior care”: Innovated mindset on intelligent senior care	Social Security Studies (Study and Practice)	2015	252
**4**	Service model problem and countermeasure of smart care for the aged in China	Social Security Studies	2017	157
**5**	Internet of things (IoT) and smart elderly care	Video Engineering	2014	152
**6**	Smart care: Innovation and thinking in the Chinese elderly care paradigm	Journal of Social Science of Hunan Normal University	2016	149
**7**	The current situation and development strategy of smart elderly care	Modern Management Science	2016	135
**8**	Effective supply and realizing a way for old-age service under PPP model	Review of Economy and Management	2017	133
**9**	Problems and countermeasures of the application of “internet plus” in community home-based endowment services	Journal of Beijing University of Posts and Telecommunications (Social Sciences Edition)	2016	126
**10**	Application and countermeasure research for information technology in the elderly care service industry	Science and Technology Management Research	2015	121

**Table 4:** The 10 most frequently cited articles from WOS.

Rank	Title	Journal	Year	Citation Count
**1**	A survey on ambient-assisted living tools for older adults	IEEE Journal of Biomedical and Health Informatics	2013	606
**2**	Cloud-assisted industrial IoT-enabled framework for health monitoring	Computer Networks	2016	377
**3**	Wireless sensor network based home monitoring system for wellness determination of the elderly	IEEE Sensor Journal	2012	208
**4**	Elderly activities recognition and classification for applications in assisted living	Expert Systems with Applications	2013	201
**5**	Design and evaluation of a smart home voice interface for the elderly: Acceptability and objection aspects	Personal and Ubiquitous Computing	2013	197
**6**	A posture recognition-based fall detection system for monitoring an elderly person in a smart home environment	IEEE Transactions on Information Technology in Biomedicine	2012	178
**7**	Forecasting the behavior of the elderly using wireless sensors data in a smart home	Engineering Applications of Artificial Intelligence	2013	170
**8**	The role of healthcare robots for older people at home: A review	International Journal of Social Robotics	2014	168
**9**	User profiles and personas in the design and development of consumer health technologies	International Journal of Medical Informatics	2013	153
**10**	A depth video sensor-based life-logging human activity recognition system for elderly care in smart indoor environments	Sensors	2014	151

**Table 5:** The most important authors in CNKI and WOS.

CNKI				WOS			
Rank	Author	Quantity	Year	Rank	Author	Quantity	Year
**1**	ZHU Q	8	2016	1	KIM J	7	2013
**2**	SUI D	8	2016	2	LEE S	6	2016
**3**	ZHANG B	6	2018	3	LI L	5	2020
**4**	XIAO M	4	2021	4	ZHANG Y	5	2020
**5**	SU W	4	2021	5	ZHANG X	4	2021
**6**	PENG Q	4	2016	6	PARK S	4	2013
**7**	ZHAO Q	4	2021	7	XU L	4	2021
**8**	XIANG Y	3	2016	8	CHEN C	3	2012
**9**	HUANG H	3	2021	9	CHEN H	3	2015
**10**	BAI M	3	2016	10	LI J	3	2022

**Table 6:** The institution networks of studies from CNKI.

Rank	Research institute	Quantity	Year
**1**	School of Information Management, NJU	10	2016
**2**	International Business School, Shanxi Normal University (IBSS, SNNU)	6	2016
**3**	School of Philosophy and Sociology, Jilin University	5	2018
**4**	Law School, Zhongnan University of Economics and Law	4	2021
**5**	Hunan Normal University	4	2015
**6**	School of Management and Engineering, NJU	4	2016
**7**	Business School, Northeast Normal University	3	2018
**8**	School of Public and Administration, NAU	3	2021
**9**	First Affiliated Hospital of Chongqiong Medical University	3	2021
**10**	Central South Normal University	3	2020

**Table 7:** Top 10 institutions for publications from WOS.

Rank	Research Institute	Quantity	Year
**1**	Hong Kong Polytech University	6	2017
**2**	Chinese Academy of Science	6	2012
**3**	Kyung Hee University	6	2012
**4**	Chang Gung University	5	2012
**5**	Deakin University	4	2017
**6**	Chaoyang University of Technology	4	2016
**7**	Yonsei University	4	2013
**8**	Zhejiang University	4	2018
**9**	Northwestern Polytechnic University	4	2015
**10**	Imperial College, London	4	2021

**Table 8:** High-frequency keywords.

CNKI			WOS		
Keywords	Count	Centrality	Keywords	Count	Centrality
Smart elderly care	16	0.19	Health care	88	0.17
Smart pension	16	0.23	System	86	0.15
AI	12	0.23	Smart home	81	0.13
Population ageing	11	0.33	Older adult	80	0.11
Elderly care service	9	0.06	Elderly care	70	0.17
Big data	7	0.09	Ambient assisted living	53	0.16
Intelligent pension	6	0.05	IoT	52	0.08
Pension service	6	0.04	Activity recognition	50	0.07
Smart senior care	6	0.05	Technology	49	0.21
Influencing factor	5	0.02	Model	23	0.11
Smart community	4	0.02	Adoption	22	0.09
Home-based care	4	0.02	Fall detection	18	0.10
Information technology	4	0.02	Impact	15	0.09
Pension industry	4	0.01	Information technology	15	0.03
Internet plus	3	0.00	Deep learning	15	0.03

**Table 9:** Burst terms in studies from CNKI and WOS.

CNKI			WOS		
Keywords	Strength	Year	Keywords	Strength	Year
Home care	1.9	2014–2015	Assistive technology	2.1	2011–2014
IoT	1.54	2017–2018	Ambient intelligence	2.66	2013–2016
Smart community	1.16	2018–2020	Wireless sensor network	2.59	2013–2015
Intelligent pension	1.61	2019–2020	Aware	2.13	2014–1026
Home care service	1.15	2019–2020	Tracking	2.54	2017–1018
Intelligent old-age care	1.41	2020–2022	Home	2.49	2017–2018
AI	1.3	2020–2022	Senior citizen	2.83	2020–2022
Home-based care	1.13	2020–2022	Population	2.12	2020–2022

## Data Availability

The data presented in this study are openly available in FigShare at https://figshare.com/articles/dataset/Smart_Elderly_Care_Review_Data/21786005 (accessed on 5 January 2023).
